# ﻿A new species of the *Cyrtodactylusbrevipalmatus* group (Squamata, Gekkonidae) from Tak Province, northwestern Thailand

**DOI:** 10.3897/zookeys.1164.101263

**Published:** 2023-05-29

**Authors:** Siriwadee Chomdej, Chatmongkon Suwannapoom, Waranee Pradit, Apichaya Phupanbai, L. Lee Grismer

**Affiliations:** 1 Department of Biology, Faculty of Science, Chiang Mai University, Chiang Mai 50200, Thailand; 2 Research Center in Bioresources for Agriculture, Industry and Medicine, Department of Biology, Faculty of Science, Chiang Mai University, Chiang Mai 50200, Thailand; 3 Division of Fishery, School of Agriculture and Natural Resources, University of Phayao, Phayao 56000, Thailand; 4 Herpetology Laboratory, Department of Biology, La Sierra University, Riverwalk Parkway, Riverside, California 92505, USA; 5 San Diego Natural History Museum, San Diego, USA

**Keywords:** Bent-toed gecko, conservation, Indochina, integrative taxonomy, Southeast Asia, systematics

## Abstract

An integrative taxonomic analysis was used to delimit and diagnose a new species of the *Cyrtodactylusbrevipalmatus* group from Tak Province in western Thailand. Although Bayesian phylogenetic analyses place *C.denticulatus***sp. nov.** within the *brevipalmatus* group, the new species is neither nested within nor is it the sister species of any other species in the *brevipalmatus* group. Furthermore, based on the mitochondrial NADH dehydrogenase subunit 2 gene (ND2) and adjacent tRNAs, it bears an uncorrected pairwise sequence divergence of 7.87–21.94% from all other species in the *brevipalmatus* group. *Cyrtodactylusdenticulatus***sp. nov.** is differetiated from all other species in the *brevipalmatus* group by having a number of unique charateristics such as denticulate ventrolateral body folds and ventrolateral subcaudal ridges, characters not seen in any other species of the group (*n* = 51 individuals). Additionally, based on a multiple factor anlaysis, *C.denticulatus***sp. nov.** does not overlap with any other species in multivariate space. The discovery of *C.denticulatus***sp. nov.** underscores the unrealized diversity of upland ecosystems across Thailand and the urgent need for increased exploration and conservation of these unique imperiled montane refugia, especially in this era of climate change.

## ﻿Introduction

The *Cyrtodactylusbrevipalmatus* group (sec. Grismer et al. 2021a, b) of Indochina and northern Sundaland has recently become the focus of a number of systematic reviews (Grismer et al. 2021c, 2022a, b, 2023; Le et al. 2021) following more than a decade-long hiatus of investigation (Grismer 2008). Grismer et al. (2022b) demonstrated that the gross underestimate of this group’s biodiversity resulted from a lack of phylogenetic data which forced the conflation of morphological diagnoses and species delimitations. Doing so resulted in the erroneous placement of 11 currently recognized species (sec. Grismer et al. 2022b) in the synonymy of just two species, *C.interdigitalis* Ulber (Ulber 1993) and *C.brevipalmatus* (Smith). The *brevipalmatus* group as currently constituted extends from northern Vietnam through Laos and central Thailand and southward along the Thai-Malay Peninsula to southern Peninsular Malaysia (Grismer et al. 2021a). Nearly all species of this group are specialized for an arboreal lifestyle and bear a prehensile tail carried in a tightly coiled position, cryptic color patterns of different shades of brown, closely matching the vegetative substrate upon which they frequent, and generally slow, deliberate “chameleon-like” movements (Grismer et al. 2020).

**Figure 1.**
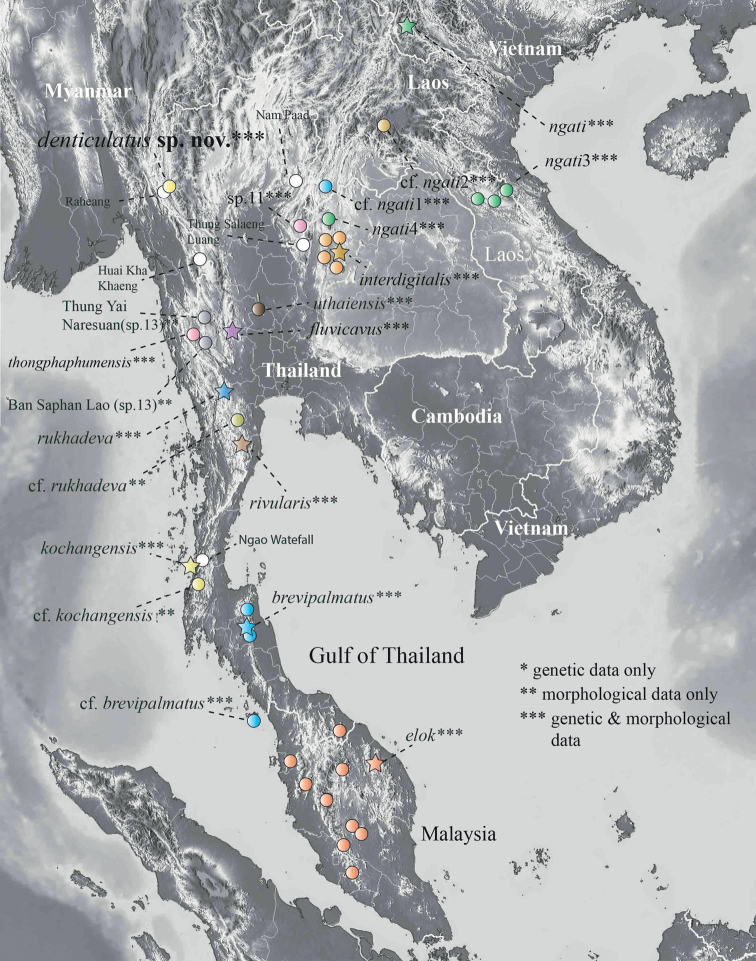
Distribution of the species of the *Cyrtodactylusbrevipalmatus* group and the localities of the specimens used in this analysis. Stars denote type localities and white circles represent the localities of unsampled populations photographed in social media.

We present herein the description of another new species of the *brevipalmatus* group from the Chao Doi waterfall, Mae Meoi, Tha Song Yang District, Tak Province, Thailand (Fig. 1). The existence of this species was first reported by Chomdej et al. (2021) in a molecular phylogeny of Thai *Cyrtodactylus* but due to a lack of morphological data, it was not described but reported as *Cyrtodactylus* sp. 10. This nomen was followed in subsequent works by Grismer et al. (2021c, 2022a, b, 2023). We now have morphological data from that specimen and comparing these data to those of all other species in the *brevipalmatus* group (*n* = 51 individuals) revealed it possesses a suite of unique morphological characters putatively separating it from all other species in the group. Based on these data, the most recent phylogeny of the group (Grismer et al. 2023), and an uncorrected pairwise sequence divergence from all other species ranging from 7.87–21.94% based on the mitochondrial NADH dehydrogenase subunit 2 gene (ND2) and adjacent tRNAs, we hypothesize this specimen represents a new species and thus we describe it herein.

## ﻿Materials

### ﻿Species delimitation

Under the general lineage concept (GLC: de Queiroz 2007) adopted herein, the molecular phylogenies recovered monophyletic mitochondrial lineages of individuals (populations) that were used to develop initial species-level hypotheses, equivalent to the grouping stage of Hillis (2019). A multivariate analysis of morphometric, meristic, and categorical data were then used to search for characters and morphospatial patterns consistent with the tree-designated species-level hypotheses (the construction of boundaries representing the hypothesis-testing step of Hillis 2019) thus providing independent diagnoses to complement the delimitations of the molecular analyses. In this way, delimiting (phylogeny) and diagnosing (taxonomy) species are not conflated (Frost and Hillis 1990; Frost and Kluge 1994; Hillis 2019).

### ﻿Genetic data

Methods for DNA extraction, sequencing, and editing followed Grismer et al. (2021c) and resulted in a 1,386 base pair segment of the mitochondrial NADH dehydrogenase subunit 2 gene (ND2) and adjacent tRNAs. All material examined and GenBank accession numbers are listed in Table 1 of Grismer et al. (2022b). The GenBank accession number for *Cyrtodactylus* sp. 10 is MT468902.

**Table 1.** Mean (minimum–maximum) percentages of uncorrected pairwise sequence divergence (*p*-distances) among the putative species of the *Cyrtodactylusbrevipalmatus* group based on 1,386 base pairs of mitochondrial NADH dehydrogenase subunit 2 gene (ND2) and adjacent tRNAs. Intraspecific p-distance are in bold font.

	*brevipalmatus*	cf.ngati1	cf.ngati2	*elok*	*fluvicavus*	*interdigitalis*	*kochangensis*	*ngati*	*rivularis*	*rukhadeva*	*thongphaphumensis*.	*denticulatus* sp. nov.	sp. 11	cf.brevipalmatus	*uthaiensis*
*brevipalmatus*	**n/a**														
*n* = 1															
cf.ngati1	21.03	**n/a**													
*n* = 1															
cf.ngati2	21.68	4.39	**n/a**												
*n* = 1															
*elok*	20.77	22.58	21.42	**n/a**											
*n* = 1															
*fluvicavus*	18.86	10.64	11.02	20.15	**0.10**										
*n* = 7	(18.84–18.97)	(10.58–10.84)	(10.97–11.23)	(20.13–20.26)	**(0.00–0.26)**										
*interdigitalis*	20.77	6.97	9.16	22.84	12.02	**n/a**									
*n* = 1					(12.00–12.13)										
*kochangensis*	19.35	14.58	14.71	20.90	12.31	15.23	**n/a**								
*n* = 1					(12.26–12.31)										
*ngati*	20.70	3.30	3.71	21.11	11.34	8.13	14.58	**0.84**							
*n* = 7	(20.65–20.90)	(2.84–4.00)	(3.35–4.26)	(20.90–21.42)	(11.10–11.87)	(7.74–8.65)	(14.45–14.84)	**(0.00–1.55)**							
*rivularis*	20.00	15.87	15.03	21.61	12.57	15.48	12.26	15.03	**0.52**						
*n* = 2	(19.74–20.26)	(15.61–16.13)	(14.84–15.23)	(21.42–21.81)	(12.26–13.03)	(15.23–15.74)	(12.00–12.52)	(14.71–15.48)							
*rukhadeva*	20.65	15.42	15.48	21.61	12.25	16.00	13.10	15.23	4.65	**1.55**					
*n* = 2	(20.13–21.16)	(14.84–16.00)	(14.84–16.13)	(21.16–22.06)	(11.61–13.03)	(15.35–16.65)	(12.52–13.68)	(14.19–16.23)	(3.61–5.68)						
*thongphaphumensis*	20.34	7.93	9.51	22.02	9.75	8.96	13.22	8.81	13.12	13.25	**0.22**				
*n* = 9	(20.13–20.65)	(7.74–8.00)	(9.42–9.55)	(21.81–22.32)	(9.55–9.94)	(8.77–9.03)	(13.03–13.29)	(8.13–9.68)	(12.77–13.42)	(12.52–13.94)	**(0.00–0.52)**				
*denticulatus* sp. nov.	19.87	9.29	10.84	21.94	10.12	10.19	13.68	10.21	13.94	14.32	8.06	**n/a**			
*n* = 1					(10.06–10.32)			(10.06–10.45)	(13.68–14.19)	(13.68–14.97)	(7.87–8.13)				
sp. 11	20.39	7.23	8.90	22.19	11.12	3.87	14.58	8.28	15.35	15.61	8.96	10.45	**n/a**		
*n* = 1					(11.10–11.23)			(8.00–8.65)	(15.10–15.61)	(14.97–16.26)	(8.77–9.03)				
cf.brevipalmatus	6.45	20.90	20.65	20.00	18.34	20.13	19.10	20.52	19.74	20.00	19.60	18.84	19.61	**n/a**	
*n* = 1					(18.32–18.45)			(20.26–20.65)	(19.48–20.00)	(19.48–20.52)	(19.48–19.87)				
*uthaiensis*	19.74	5.81	8.13	21.16	10.12	7.1	13.94	6.97	13.94	13.94	7.80	8.39	6.58	19.48	**n/a**
*n* = 1					(10.06–10.32)			(6.58–7.61)	(13.68–14.19)	(13.29–14.58)	(7.61–7.87)				

### ﻿Morphological data

The morphological data included 15 meristic, 16 normalized morphometric, and eight categorical characters. The data were taken using the protocol of Le et al. (2021) and Grismer et al. (2022a). All data were taken on the left side of the body (when possible) and morphometric characters were measured to the nearest 0.1 mm using digital calipers under a Nikon SMZ745 stereomicroscope. Morphometric data taken were: snout-vent length (**SVL**), taken from the tip of the snout to the vent; tail length (**TL**), taken from the vent to the tip of the tail, original or partially regenerated; tail width (**TW**), taken at the base of the tail immediately posterior to the postcloacal swelling (TL and TW were too variable between sexes and condition of the tail to be used in the analyses); humeral length (**HumL**), taken from the proximal end of the humerus at its insertion point in the glenoid fossa to the distal margin of the elbow while flexed 90°; forearm length (**ForL**), taken on the ventral surface from the posterior margin of the elbow while flexed 90° to the inflection of the flexed wrist; femur length (**FemL**), taken from the proximal end of the femur at its insertion point in the acetabulum to the distal margin of the knee while flexed 90°; tibia length (**TibL**), taken on the ventral surface from the posterior margin of the knee while flexed 90° to the base of the heel; axilla to groin length (**AG**), taken from the posterior margin of the forelimb at its insertion point on the body to the anterior margin of the hind limb at its insertion point on the body; head length (**HL**), the distance from the posterior margin of the retroarticular process of the lower jaw to the tip of the snout; head width (**HW**), measured at the angle of the jaws; head depth (**HD**), the maximum height of head measured from the occiput to base of the lower jaw posterior to the eyes; eye diameter (**ED**), the greatest horizontal diameter of the eye-ball; eye to ear distance (**EE**), measured from the anterior edge of the ear opening to the posterior edge of the bony orbit; eye to snout distance or snout length (**ES**), measured from anteriormost margin of the bony orbit to the tip of snout; eye to nostril distance (**EN**), measured from the anterior margin of the bony orbit to the posterior margin of the external nares; interorbital distance (**IO**), measured between the dorsomedial-most edges of the bony orbits; internarial distance (**IN**), measured between the external nares across the rostrum; and ear length (**EL**), greatest oblique length across the auditory meatus. Normalization of the morphometric characters in order to prevent allometric biasing follows Chan and Grismer (2022).

Evaluated meristic characters were the number of supralabial scales (**SL**), counted from the largest scale at the corner of the mouth or posterior to the eye, to the rostral scale; infralabial scales (**IL**), counted from termination of enlarged scales at the corner of the mouth to the mental scale; number of paravertebral tubercles (**PVT**) between the limb insertions counted in a straight line immediately left of the vertebral column; number of longitudinal rows of body tubercles (**LRT**) counted transversely across the body midway between the limb insertions from one ventrolateral body fold to the other; number of longitudinal rows of ventral scales (**VS**) counted transversely across the abdomen midway between limb insertions from one ventrolateral fold to the other; number of transverse rows of ventral scales (**VSM**)counted along the midline of the body from the postmentals to just anterior to the cloacal opening, stopping where the scales become granular; number of expanded subdigital lamellae on the fourth toe proximal to the digital inflection (**TL4E**) counted from the base of the first phalanx where it contacts the body of the foot to the largest scale on the digital inflection; the large contiguous scales on the palmar and plantar surfaces were not counted; number of small, generally unmodified subdigital lamellae distal to the digital inflection on the fourth toe (**TL4U**)counted from the digital inflection to the claw including the claw sheath; total number of subdigital lamellae (**TL4T**) beneath the fourth toe (i.e. TL4E + TL4U = TL4T); number of expanded subdigital lamellae on the fourth finger proximal to the digital inflection (**FL4E**) counted the same way as with TL4E; number of small generally unmodified subdigital lamellae distal to the digital inflection on the fourth finger (**FL4U**) counted the same way as with TL4U; total number of subdigital lamellae (**FL4T**) beneath the fourth toe (i.e. FL4E + FL4U = FL4T); total number of enlarged femoral scales (**FS**) from each thigh combined as a single metric; number of enlarged precloacal scales (**PCS**); number of precloacal pores (**PP**) in males; number of femoral pores (**FP**) in males from each thigh combined as a single metric; number postcloacal tubercles (**PCT**) on each side of the base of the tail (this character was not used in the analyses); and the number of dark body bands (**BB**) between the dark band on the nape and the hind limb insertions on the body. A post-sacral or sacral band when present, was not counted. Categorical characters evaluated were the presence or absence of tubercles on the flanks (**FKT**); ventrolateral body fringe denticulate (**VFD**); slightly enlarged medial subcaudals (**SC1**); single enlarged, unmodified, medial subcaudal scales (**SC2**); enlarged medial subcaudals intermittent, medially furrowed, posteriorly emarginated (**SC3**); small longitudinal ventrolateral subcaudal ridge (**SC4**); large or small dorsolateral caudal tubercles (**DCT**)forming a narrow or wide ventrolateral caudal fringe (**VLF1**); ventrolateral caudal fringe scales generally homogenous or not (**VLF2**); and the cross-section of the tail round or square (**TLcross**).

### ﻿Phylogenetic analyses

An input file implemented in BEAUti (Bayesian Evolutionary Analysis Utility) v. 2.4.6 was run in BEAST (Bayesian Evolutionary Analysis Sampling Trees) v. 2.4.6 (Drummond et al. 2012) on CIPRES (Cyberinfrastructure for Phylogenetic Research; Miller et al. 2010) in order to generate a BEAST phylogeny. Data were partitioned by codon and a lognormal relaxed clock with unlinked site models and linked trees and clock models were employed. bModelTest (Bouckaert and Drummond 2017), implemented in BEAST, was used to numerically integrate over the uncertainty of substitution models while simultaneously estimating phylogeny using Markov chain Monte Carlo (MCMC). MCMC chains were run using a birth-death prior for 40,000,000 million generations and logged every 4,000 generations. The BEAST log file was visualized in Tracer v. 1.7.0 (Rambaut et al. 2018) to ensure effective sample sizes (ESS) were well-above 200 for all parameters. A maximum clade credibility tree using mean heights at the nodes was generated using TreeAnnotator v. 1.8.0 (Rambaut and Drummond 2013) with a burn-in of 1,000 trees (10%). Nodes with Bayesian posterior probabilities (BPP) of 0.95 and above were considered strongly supported (Huelsenbeck et al. 2001; Wilcox et al. 2002). Uncorrected pairwise sequence divergences were calculated in MEGA 11 (Tamura et al. 2021) using the complete deletion option to remove gaps and missing data from the alignment prior to analysis.

### ﻿Statistical analyses

All statistical analyses were conducted using R Core Team (2018). Morphometric characters used in statistical analyses were SVL, AG, HumL, ForL, FemL, TibL, HL, HW, HD, ED, EE, ES, EN, IO, EL, and IN. Tail metrics were not used due to the high degree of incomplete sampling (i.e,. regenerated, broken, or missing). In order to most successfully remove the effects of allometry (sec. Chan and Grismer 2022), size was normalized using the following equation: X_adj_ = log(X)-β[log(SVL)-log(SVL_mean_)], where X_adj_ = adjusted value; X = measured value; β = unstandardized regression coefficient for each population; and SVL_mean_ = overall average SVL of all populations (Thorpe 1975, 1983; Turan 1999; Lleonart et al. 2000), accessible in the R package GroupStruct (available at https://github.com/chankinonn/GroupStruct). The morphometrics of each species were normalized separately and then concatenated so as not to conflate potential intra- with interspecific variation (Reist 1986; McCoy et al. 2006). The juvenile *Cyrtodactylusngati* (HNUE-R00112) was removed from the data so as not to skew the normalization results. All data were scaled to their standard deviation to ensure they were analyzed on the basis of correlation and not covariance. Meristic characters analyzed were SL, IL, PVT, LRT, VS, VSM, TL4E, TL4U, TL4T, FL4E, FL4U, FL4T, FS, PCS, and BB. Precloacal and femoral pores were omitted from the multivariate analyses due to their absence in females. Categorical characters analyzed were DCT, VLF1, VLF2, TLcross, SC1, SC2, and SC3.

Morphospatial clustering and positioning among the species/populations and individuals was analyzed using multiple factor analysis (MFA) on a concatenated data set comprised of 15 meristic characters, 16 normalized morphometric characters, and eight categorical characters (Suppl. material 1). The MFA was implemented using the *mfa* () command in the R package FactorMineR (Husson et al. 2017) and visualized using the Factoextra package (Kassambara and Mundt 2017). MFA is a global, unsupervised, multivariate analysis that incorporates qualitative and quantitative data (Pagès 2015), making it possible to analyze different data types simultaneously in a nearly total evidence environment. In an MFA, each individual is described by a distinct set of variables (i.e., characters) which are structured into different data groups in a global data frame, in this case, quantitative data (i.e., meristics and normalized morphometrics) and categorical data (i.e., scale, tubercle, and caudal morphology). In the first phase of the analysis, separate multivariate analyses are conducted for each set of variables: principal component analyses (PCA) for each quantitative data set and a multiple correspondence analysis (MCA) for the categorical data. The data sets are then normalized separately by dividing all their elements by the square root of their first eigenvalues. For the second phase of the analysis, these normalized data sets are concatenated into a single matrix for a global PCA of the normalized data. Standardizing the data in this manner prevents one data type from overleveraging another. In other words, the normalization of the data in the first phase prevents data types with the highest number of characters or the greatest amount of variation from outweighing other data types in the second phase. This way, the contribution of each data type to the overall variation in the data set is scaled to define the morphospatial distance between individuals as well as calculating each data type’s contribution to the overall variation in the analysis (Pagès 2015; Kassambara and Mundt 2017).

## ﻿Results

### ﻿Phylogenetic analysis

**Figure 2.**
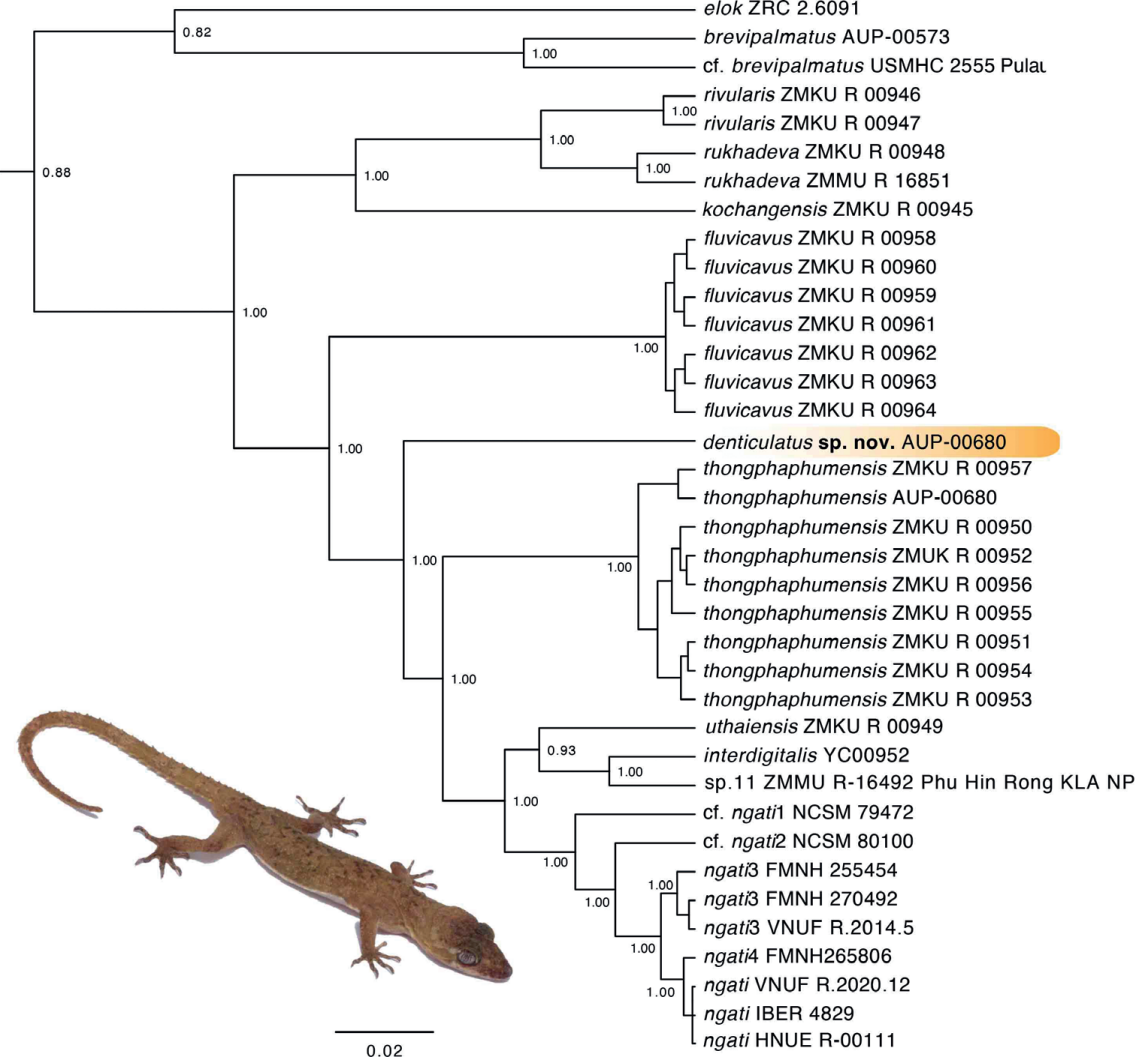
Maximum clade credibility BEAST phylogeny of the *Cyrtodactylusbrevipalmatus* group highlighting the new species described herein. Bayesian posterior probabilities (BPP) are listed at the nodes.

The BEAST analysis recovered *C.* sp. 10 as being deeply nested within the *brevipalmatus* group on a long branch that was not embedded within, nor sister to any other species (Fig. 2). *Cyrtodactylus* sp. 10 was strongly recovered (BPP 1.00) recovered as the sister species to a lineage composed of *Cyrtodactylusthongphaphumensis*, *C.uthaiensis*, *C.interdigitalis*, *C.* sp. 11, C.cf.ngati1, C.cf.ngati2, *C.ngati*3, *C.ngati*4, and *C.ngati*. *Cyrtodactylus* sp. 10 and has an uncorrected pairwise sequence divergence from all other species ranging from 7.87–21.94% (Table 1).

### ﻿Multiple factor analysis

**Figure 3.**
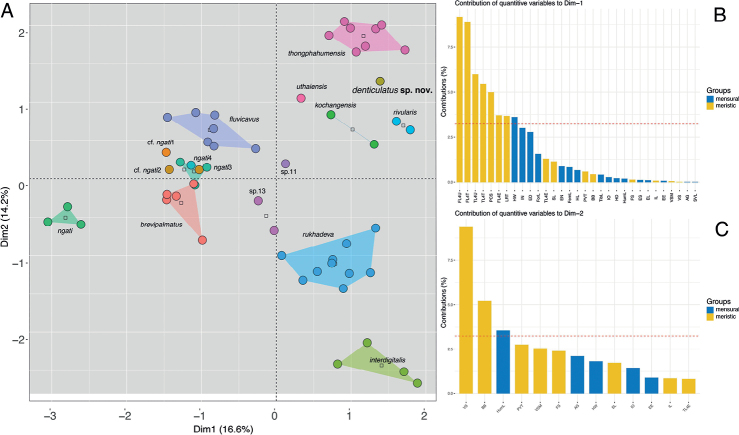
MFA analyses of the *Cyrtodactylusbrevipalmatus* group **A**MFA of the species-level lineages based on the BEAST phylogeny (Fig. 2) **B** percent contribution of morphometric and meristic characters to Dimension 1 of the MFA. The red dotted line represents the average contribution **C** percent contribution of morphometric and meristic characters to Dimension 2 of the MFA. The red dotted line represents the average contribution.

The MFA recovered *C.* sp. 10 as well-separated from all other species of the *brevipalmatus* group along the ordination of the first two dimensions (Dim) in that the specimen did not cluster near or within the convex hull of any other species (Fig. 3A). Dim-1 accounted for 16.6% of the variation in the data set and loaded most heavily for the meristic characters FL4U, FL4T, TL4U, TL4T, PCS, FL4E, LRT, and the morphometric character HW in that they contributed a greater than average amount of variation along Dim-1 (Fig. 3B). The meristic characters VS and BB and the morphometric character HumL contributed a greater than average amount of variation along Dim-2 (Fig. 3C). Dim-1 and Dim-2 collectively accounted for a total of 38% of the variation in the data set. The first five dimensions account for a total of 60.7% of the variation in the data set.

## ﻿Taxonomy

Based on the phylogenetic position of *C.* sp. 10, its high percentage value of pairwise sequence divergence from all other species, and its unique morphology, we hypothesize *C.* sp. 10 represents a diagnosable evolutionarily distinct population at the northwestern extent of the range of the *brevipalmatus* group that should be recognized as a new species. As such, it is described below.

### ﻿ Cyrtodactylus denticulatus sp. nov.

Taxon classificationAnimaliaSquamataGekkonidae

D17C3A1D-7423-538B-B521-4802DBAF8B32

https://zoobank.org/5052D212-71D3-43D5-B324-01F8BE8DF56C

**Figure 4.**
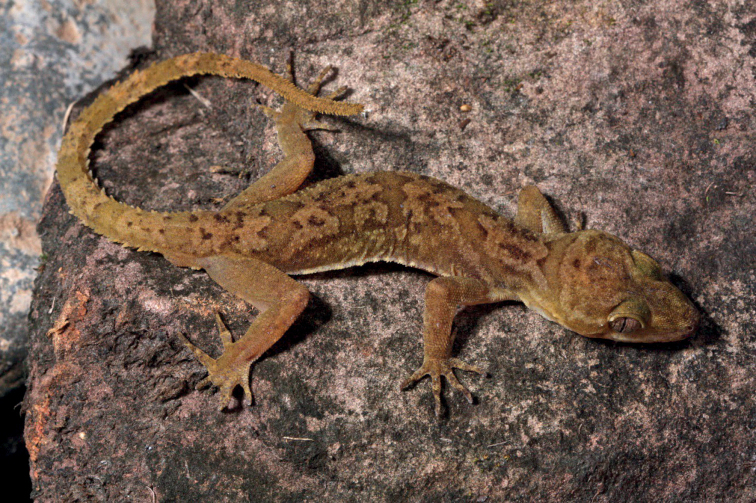
Photograph of the living holotype of *Cyrtodactylusdenticulatus* sp. nov. AUP-00680 from the Chao Doi waterfall, Mae Meoi, Tha Song Yang District, Tak Province, Thailand.

**Figure 5.**
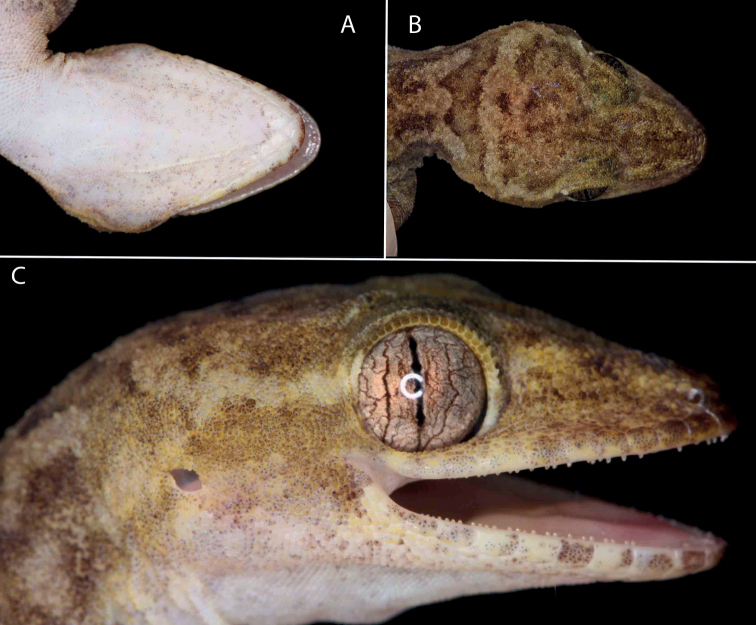
Head of the holotype of *Cyrtodactylusdenticulatus* sp. nov. AUP-00680 from the Chao Doi waterfall, Mae Meoi, Tha Song Yang District, Tak Province, Thailand **A** ventral view **B** dorsal view **C** right lateral view.

**Figure 6.**
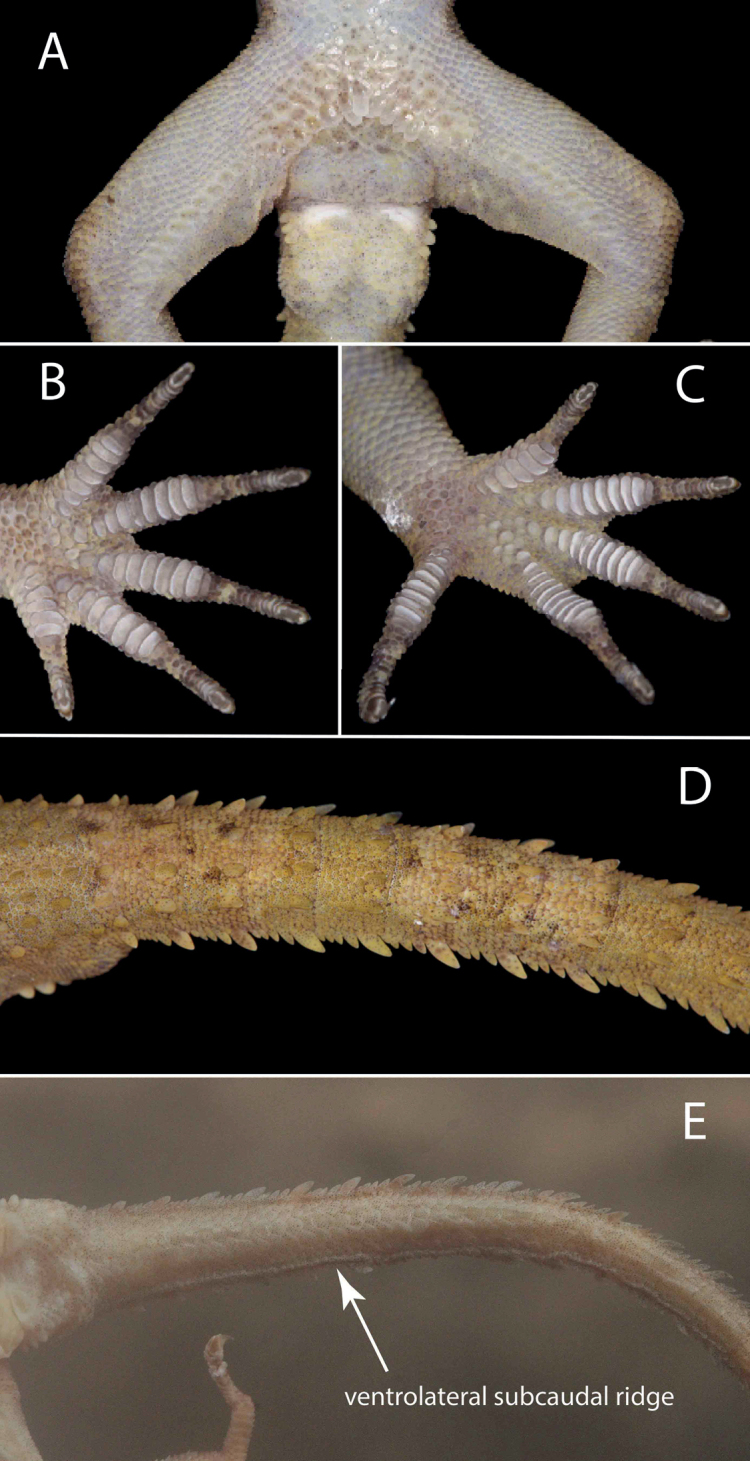
Holotype of *Cyrtodactylusdenticulatus* sp. nov. AUP-00680 from the Chao Doi waterfall, Mae Meoi, Tha Song Yang District, Tak Province, Thailand **A** ventral view of the cloacal and femoral regions **B** ventral view of the left hand **C** ventral view of the left foot **D** dorsal view of the anterior section of the tail **E** ventral view of the tail showing the ventrolateral subcaudal ridge.

Figs 4 , 5 , 6 Suggested common name: Spiny-tailed bent-toed gecko Suggested Thai common name: ตุ๊กกายฟันเลื่อย Tuk Kay Fhun Leuy

Cyrtodactylus sp. 10: Chomdej et al. 2021: 11; Grismer et al. 2021: 725, 2022a: 247, 2022b: 111; 2023: 95.

#### Type material.

***Holotype*.** Adult male AUP-00680 collected on 8 March 2019 by Parinya Pawangkhanant and Chatmongkon Suwannapoom from a bamboo forest near a rocky stream at the Chao Doi waterfall, Mae Meoi, Tha Song Yang District, Tak Province, Thailand at 17°30.000'N, 98°03.000'E (DDM) and 610 m a.s.l.

#### Diagnosis.

*Cyrtodactylusdenticulatus* sp. nov. is tentatively separated (see below) from all other species of the *brevipalmatus* group by the combination of having nine supralabials, nine infralabials, 20 paravertebral tubercles, 19 rows of longitudinally arranged tubercles, 42 transverse rows of ventrals, 158 longitudinal rows of ventrals, nine expanded subdigital lamellae on the fourth finger, 11 unexpanded subdigital lamellae on the fourth toe, 19 total subdigital lamellae on the fourth toe; nine expanded subdigital lamellae on the fourth finger, ten unexpanded subdigital lamellae on the fourth finger, 19 total subdigital lamellae on the fourth finger; 16 total number of enlarged femoral scales, 20 total number of femoral pores in the male specimen; 13 precloacal pores in the male specimen; 16 enlarged precloacals; enlarged femorals and enlarged precloacals not continuous; proximal femorals smaller than distal femorals; tubercles on forelimbs and flanks nearly same size as those on body; ventrolateral body fold weakly denticulate; spinose paravertebral rows; row of large dorsolateral caudal tubercles; wide ventrolateral caudal fringes; ventrolateral caudal fringes composed scales of different size; small longitudinal ventrolateral subcaudal ridges; tail square in cross-section; no slightly enlarged unpaired medial subcaudals; subcaudals not posteromedially furrowed; SVL 69.5 mm; three dark transverse body bands (Tables 2–4).

**Table 2.** Sex, raw meristic, categorical, and morphometric data used in the analyses of specimens in the *Cyrtodactylusbrevipalmatus* group. ♂ = male; ♀ = female; R/L = right/left; / = data unavailable. Shaded cells denote characters that potentially differentiate *C.denticulatus* sp. nov. from the other species.

Species	*denticulatus* sp. nov.	*brevipalmatus*	cf.brevipalmatus	cf.brevipalmatus	*brevipalmatus*	*brevipalmatus*	*elok*	*elok*	*elok*	*elok*	*interdigitalis*	*interdigitalis*	*interdigitalis*	*interdigitalis*	sp. 11
Institutional catalog number	AUP-00680	LSUHC 1899	LSUHC 15076	LSUHC 11788	THNHM 10670	THNHM 14112	LSUHC 8238	LSUHC 12180	LSUHC 12181	ZMMU R-16144	THNHM 20226 paratype	THNHM 20228 paratype	THNHM 20229 paratype	THNMH 20227 paratype	ZMMU R-16492
Sex	♂	♂	♀	♀	♀	♀	♀	♂	♂	♀	♀	♀	♀	♀	♂
**Meristic data**															
supralabials (SL)	9	11	12	10	14	12	11	8	13	9	14	12	11	12	11
infralabials (IL)	9	8	10	9	11	11	11	8	11	9	9	8	8	7	9
paravertebral tubercles (PVT)	20	39	37	38	37	37	0	0	0	0	32	33	33	33	30
longitudinal rows of tubercles (LRT)	19	15	16	17	16	14	6	7	4	4	19	20	19	19	18
ventral scales (VS)	42	38	38	38	36	39	45	45	47	36	42	40	42	43	34
ventral scales along middle of the body (VSM)	158	176	170	182	154	160	190	225	234	192	187	170	187	178	160
expanded subdigital lamellae on 4^th^ toe (TL4E)	9	7	8	9	8	8	10	9	9	9	12	10	10	11	9
unmodified subdigital lamellae on 4^th^ toe (TL4U)	11	13	11	11	11	12	11	10	11	9	14	13	12	14	10
total subdigital lamellae 4^th^ toe (TL4T)	20	20	19	20	19	20	21	19	20	18	26	23	22	23	19
expanded subdigital lamellae on 4^th^ finger (FL4E)	9	8	8	8	7	8	8	9	9	9	9	8	9	9	10
unmodified subdigital lamellae on 4^th^ finger (FL4U)	10	9	11	10	10	10	12	13	9	8	12	11	12	13	9
total subdigital lamellae 4^th^ finger (FL4T)	19	17	19	18	17	18	20	22	18	17	21	21	21	22	19
enlarged femoral scales (R/L)	8	0	0	0	8R/8L	7R/7L	0	0	0	0	11R/8L	10R/9L	8R/8L	9R/10L	9R/8L
total enlarged femoral scales (FS)	16	16	10	11	16	14	0	0	0	0	14	19	19	19	17
total femoral pores in males (FP)	20	7	/	/	/	/	/	0	0	/	/	/	/	/	17
enlarged precloacal scales (PCS)	13	7	7	7	8	7	8	8	8	7	14	15	13	19	13
precloacal pores in males (PP)	13	7	/	/	/	/	/	8	8	/	0	/	0	0	13
postcloacal tubercles (PCT)	3	3	3	2	3	3	3	2	3	3	3	2	3	3	3
Body bands (BB)	3	4	6	3	5	5	5	5	3	3	5	5	5	5	3
**Categorical data**															
small tubercles on flank (FKT)	present	present	present	present	present	present	absent	absent	absent	absent	present	present	present	present	present
denticulate ventrolateral body folds (VFD)	present	absent	absent	absent	absent	absent	absent	absent	absent	absent	absent	absent	absent	absent	absent
dorsolateral caudal tubercles (DCT)	small	small	small	small	/	small	large	large	large	large	small	/	small	small	large
ventrolateral caudal fringe narrow or wide (VLF1)	wide	narrow	narrow	narrow	/	narrow	wide	wide	wide	wide	narrow	/	narrow	narrow	wide
ventrolateral caudal fringe scales generally homogenous (VLF2)	no	no	no	no	/	no	no	no	no	no	yes	yes	yes	yes	yes
tail cross-section (TLcross)	square	circular	circular	circular	/	circular	square	square	square	square	circular	/	circular	circular	square
slightly enlarged medial subcaudals (SC1)	absent	present	present	present	/	absent	absent	absent	absent	absent	absent	/	absent	absent	present
single enlarged medial subcaudal (SC2)	absent	absent	absent	absent	/	absent	absent	absent	absent	absent	absent	/	absent	absent	absent
enlarged medial subcaudals intermittent, medially furrowed, posteriorly emarginate (SC3)	no	no	no	no	/	no	no	no	no	no	yes	/	yes	yes	no
small ventrolateral subcaudal ridge of scales (SC4)	yes	no	no	no	no	no	no	no	no	no	no	no	no	no	no
**Morphometric data**															
SVL	69.5	68.8	70.8	64.1	65.95	63.79	80.2	78.2	84.8	78.6	81.19	74.80	78.56	59.70	68.1
AG	83.0	35.7	33.4	30.1	30.0	26.5	39.7	37.8	41.5	36.2	34.5	33.7	32.7	24.6	34.6
HumL	9.1	9.7	9.3	8.0	9.6	9.5	10.2	9.1	10.1	1.7	9.8	10.2	11.2	7.4	10.3
ForL	10.2	9.9	9.8	8.9	8.2	8.7	11.5	11.7	11.8	10.2	10.6	10.5	11.1	8.4	8.5
FemL	12.8	12.0	12.6	11.5	11.7	9.8	12.9	14.2	14.6	13.1	14.7	13.2	12.7	10.2	12.6
TibL	6.5	11.6	12.2	10.5	9.7	8.2	13.5	14.0	13.8	12.3	13.1	11.9	12.9	10.2	11.4
HL	18.8	19.3	19.3	19.0	17.9	18.2	21.8	21.6	21.9	21.7	20.8	19.9	21.7	16.7	18.4
HW	12.7	13.2	13.8	12.3	12.3	12.0	15.6	16.1	15.9	15.1	14.0	13.4	14.2	11.4	13.1
HD	7.2	8.0	7.6	7.6	7.3	7.0	9.6	9.8	10.4	9.8	3.4	8.6	8.7	6.6	8.3
ED	4.0	5.2	4.5	4.3	5.3	4.4	4.8	5.0	5.7	5.0	5.3	5.5	5.9	4.4	4.4
EE	5.4	5.7	5.9	4.9	5.7	5.7	6.4	7.1	7.0	6.8	5.8	6.2	6.4	4.8	6.2
ES	7.9	7.4	7.6	7.0	7.0	7.2	8.6	8.7	9.5	8.6	8.3	7.8	9.1	6.8	7.7
EN	5.8	5.7	5.4	4.9	5.3	5.4	6.0	6.2	6.5	6.2	6.0	5.5	6.8	5.1	5.5
IO	7.1	5.4	4.7	4.7	4.2	5.2	5.7	5.4	5.4	3.9	4.8	4.7	5.5	4.3	2.9
EL	0.7	1.0	1.4	1.1	1.3	1.0	1.9	1.4	1.5	1.4	1.3	1.3	1.6	1.2	0.9
IN	2.4	1.7	2.1	2.3	2.1	2.2	2.7	2.6	2.5	3.1	2.1	2.2	2.5	1.8	2.3

**Table 3.** Sex, raw meristic, categorical, and morphometric data used in the analyses of specimens in the *Cyrtodactylusbrevipalmatus* group. ♂ = male; ♀ = female; R/L = right/left; / = data unavailable. Shaded cells denote characters that potentially differentiate *C.denticulatus* sp. nov. from the other species.

Species	*denticulatus* sp. nov.	*ngati*				*ngati*3			*ngati*4	cf.ngati1	cf.ngati2	
Institutional catalog number	AUP-00680	HNUE-R00111	IEBR 4829	VNUF R.2020.12	HNUE-R00112	FMNH 255454	FMNH 270493	FMNH 270492	FMNH 265806	NCSM 79472	ZMMU R-14917	NCSM 80100
Sex	♂	♂	♀	♀	♀	♀	♂	♂	♂	♀	♀	♀
**Meristic data**												
supralabials (SL)	9	10	10	10	10	13	13	13	10	14	9	12
infralabials (IL)	9	9	9	9	9	10	9	11	8	11	10	12
paravertebral tubercles (PVT)	20	39	40	38	40	28	27	26	27	28	32	29
longitudinal rows of tubercles (LRT)	19	18	18	17	22	19	18	17	19	18	24	19
ventral scales (VS)	42	38	36	35	32	37	36	36	33	33	36	35
ventral scales along middle of the body (VSM)	158	168	164	178	158	159	166	156	158	164	166	165
expanded subdigital lamellae on 4^th^ toe) TL4E(	9	8	10	9	9	10	10	8	10	9	8	10
unmodified subdigital lamellae on 4^th^ toe) TL4U(	11	11	10	11	10	11	11	11	11	12	10	10
total subdigital lamellae 4^th^ toe (TL$T)	20	13	16	17	16	21	21	19	21	21	18	20
expanded subdigital lamellae on 4^th^ finger (FL4E)	9	6	6	7	6	8	8	8	8	9	7	9
unmodified subdigital lamellae on 4^th^ finger (FL4U)	10	9	9	9	9	10	10	10	10	8	9	10
total subdigital lamellae 4^th^ finger (FL4T)	19	15	15	18	15	18	18	18	18	17	16	19
enlarged femoral scales (R/L)	8	10R/10L	9R/8L	10R/9L	8R/9L	9R/7L	8R/9L	9R/9L	8R/8L	9R/8L	7R/8L	7R/8L
total enlarged femoral scales (FS)	16	20	17	19	17	16	17	18	16	17	15	15
total femoral pores (FP)	20	14	/	/	/	/	14	15	13	/	/	/
enlarged precloacal scales (PCS)	13	13	13	13	13	15	13	13	13	12	13	13
precloacal pores (PP)	13	13	/	/	/	13	13	13	13	/	/	/
postcloacal tubercles (PCT)	3	3	2	1	2	/	/	/	/	2	3	4
Body bands (BB)	3	6	6	6	6	3	4	3	3	3	3	3
**Categorical data**												
small tubercles on flank (FKT)	present	present	present	present	present	present	present	present	present	present	present	present
denticulate ventrolateral body folds (VFD)	present	absent	absent	absent	absent	absent	absent	absent	absent	absent	absent	absent
dorsolateral caudal tubercles (DCT)	small	small	small	small	small	small	small	small	small	small	small	small
ventrolateral caudal fringe narrow or wide (VLF1)	wide	narrow	narrow	narrow	narrow	narrow	narrow	narrow	narrow	narrow	narrow	narrow
ventrolateral caudal fringe scales generally homogenous (VLF2)	no	no	no	no	no	yes	yes	yes	yes	yes	yes	yes
tail cross-section (TLcross)	square	circular	circular	circular	circular	circular	circular	circular	circular	circular	circular	circular
slightly enlarged medial subcaudals (SC1)	absent	present	present	present	present	/	present	present	present	present	present	present
single enlarged medial subcaudal (SC2)	absent	absent	absent	absent	absent	/	absent	absent	absent	absent	absent	absent
enlarged medial subcaudals intermittent, medially furrowed, posteriorly emarginate (SC3)	no	no	no	no	no	/	no	no	no	no	no	no
small ventrolateral subcaudal ridge of scales (SC4)	yes	no	no	no	no	/	no	no	no	no	no	no
**Morphometric data**												
SVL	69.5	66.5	68.1	69.3	46.6	83.6	70.2	74.1	73.8	78.0	87.1	77.7
AG	83.0	28.8	29.8	30.2	19.7	41.3	35.4	37.0	31.3	38.2	41.9	36.8
HumL	9.1	7.9	8.1	8.5	5.6	8.6	8.7	8.6	6.9	8.7	11.5	9.2
ForL	10.2	9.2	10.0	10.1	6.5	10.2	9.3	10.4	10.0	10.3	10.4	10.7
FemL	12.8	11.5	11.5	11.5	7.6	13.7	12.7	13.0	13.1	13.1	15.2	14.2
TibL	6.5	10.8	11.1	11.8	7.8	12.5	11.8	11.2	11.1	12.8	12.6	12.7
HL	18.8	20.1	20.4	20.7	16.1	21.7	20.6	20.3	20.7	21.2	22.1	21.4
HW	12.7	12.6	12.0	11.8	8.8	13.8	12.5	13.0	12.3	12.7	14.8	13.5
HD	7.2	7.4	7.2	6.6	5.1	9.2	8.4	9.1	7.6	8.3	8.7	9.2
ED	4.0	3.8	4.1	3.4	2.6	4.9	4.9	4.9	4.8	6.5	4.6	6.0
EE	5.4	5.8	5.5	5.9	4.4	6.9	6.1	6.2	5.7	5.3	6.5	6.2
ES	7.9	7.5	7.6	6.9	5.0	9.0	8.3	8.3	8.2	8.7	8.8	8.4
EN	5.8	6.7	6.3	6.2	4.5	6.5	6.2	6.1	6.2	6.2	6.6	6.0
IO	7.1	5.6	5.4	5.6	4.2	6.6	5.6	5.4	5.1	4.9	3.5	5.7
EL	0.7	0.8	0.8	0.7	0.3	1.3	1.1	1.2	1.0	1.5	1.2	0.9
IN	2.4	2.8	2.6	2.6	2.0	2.8	2.5	2.5	2.3	2.7	2.2	2.5

**Table 4.** Sex, raw meristic, categorical, and morphometric data used in the analyses of specimens in the *Cyrtodactylusbrevipalmatus* group. ♂ = male; ♀ = female; R/L = right/left; / = data unavailable. Shaded cells denote characters that potentially differentiate *C.denticulatus* sp. nov. from the other species.

Species	*denticulatus* sp. nov.	*thongphaphumensis*								sp.13	
Institutional catalog number	AUP-00680	ZMKU R 00950 paratype	ZMKU R 00951 paratype	ZMKU R 00952 paratype	ZMKU R 00953 holotype	ZMKU R 00954 paratype	ZMKU R 00955 pratype	ZMKU R 00956 paratype	ZMKU R 00957 paratype	THNHM 00104	THNHM 27821
Sex	♂	♀	♂	♀	♂	♂	♀	♂	♀	♀	♀
**Meristic data**											
supralabials (SL)	9	12	13	13	14	13	13	13	13	12	15
infralabials (IL)	9	8	8	10	10	9	10	10	9	10	11
paravertebral tubercles (PVT)	20	32	33	34	34	36	36	30	30	33	29
longitudinal rows of tubercles (LRT)	19	21	19	20	20	21	21	19	19	18	20
ventral scales (VS)	42	34	33	33	34	30	33	32	33	37	36
ventral scales along middle of the body (VSM)	158	173	158	156	166	159	159	150	169	159	165
expanded subdigital lamellae on 4^th^ toe (TL4E)	9	9	10	9	8	10	8	9	9	9	7
unmodified subdigital lamellae on 4^th^ toe (TL4U)	11	12	14	13	12	13	12	11	13	12	12
total subdigital lamellae 4^th^ toe (TL$T)	20	21	24	22	20	23	20	20	22	21	19
expanded subdigital lamellae on 4^th^ finger (FL4E)	9	8	7	7	8	8	8	8	8	8	8
unmodified subdigital lamellae on 4^th^ finger (FL4U)	10	10	12	12	11	12	12	11	12	11	10
total subdigital lamellae 4^th^ finger (FL4T)	19	18	19	19	19	20	20	19	20	19	18
enlarged femoral scales (R/L)	8	5R/7L	8R/8L	8R/8L	7R/8L	8R/8L	7R/8L	7R/6L	8R/8L	9R/9L	7R/10L
total enlarged femoral scales (FS)	16	12	16	16	15	16	15	13	16	18	17
total femoral pores (FP)	20	0	16	0	14	15	14	12	0	0	0
enlarged precloacal scales (PCS)	13	17	15	15	15	15	15	15	15	14	16
precloacal pores (PP)	13	0	15	0	15	15	15	15	0	0	0
postcloacal tubercles (PCT)	3	2	2R/3L	3	3	2R/3L	2R/3L	3	2	3	3
Body bands (BB)	3	3	4	3	4	3	5	4	4	3	/
**Categorical data**											
small tubercles on flank (FKT)	present	present	present	present	present	present	present	present	present	present	present
denticulate ventrolateral body folds (VFD)	present	absent	absent	absent	absent	absent	absent	absent	absent	absent	absent
dorsolateral caudal tubercles (DCT)	small	large	large	large	large	large	large	/	large	small	small
ventrolateral caudal fringe narrow or wide (VLF1)	wide	wide	wide	wide	wide	wide	wide	/	wide	narrow	narrow
ventrolateral caudal fringe scales generally homogenous (VLF2)	no	no	no	no	no	no	no	/	no	yes	yes
tail cross-section (TLcross)	square	square	square	square	square	square	square	/	square	circular	circular
slightly enlarged medial subcaudals (SC1)	absent	present	present	present	present	present	present	/	present	present	present
single enlarged medial subcaudal (SC2)	absent	absent	absent	absent	absent	absent	absent	/	absent	absent	absent
enlarged medial subcaudals intermittent, medially furrowed, posteriorly emarginate (SC3)	no	no	no	no	no	yes	no	/	no	no	no
small ventrolateral subcaudal ridge of scales (SC4)	yes	no	no	no	no	no	no	/	no	no	no
**Morphometric data**											
SVL	69.5	73.1	73.5	73.7	73.2	64.4	76.6	76.6	74.2	63.7	72.9
AG	83.0	34.8	33.9	35.4	33.6	28.5	37.1	33.2	35.1	25.8	30.6
HumL	9.1	8.4	7.2	9.0	9.0	7.2	8.0	8.1	8.6	7.6	10.1
ForL	10.2	9.5	9.1	9.2	9.8	9.2	10.0	8.6	9.8	8.1	9.6
FemL	12.8	12.8	11.6	12.3	12.5	10.9	13.7	10.8	12.5	10.7	12.8
TibL	6.5	10.5	10.1	10.6	10.6	9.9	11.1	10.0	11.4	10.1	10.2
HL	18.8	19.9	20.9	20.1	20.0	17.6	20.4	19.3	20.0	17.6	19.9
HW	12.7	14.5	14.3	15.7	13.9	12.8	14.7	14.4	14.1	11.9	13.8
HD	7.2	7.8	7.7	7.9	7.7	7.0	8.2	7.8	7.6	7.7	8.4
ED	4.0	5.0	5.1	5.0	5.0	4.8	5.6	5.3	4.9	4.1	5.3
EE	5.4	5.9	5.9	6.0	5.9	5.3	6.1	6.0	6.0	4.9	6.3
ES	7.9	7.9	8.5	7.9	7.9	7.3	8.2	7.9	7.9	7.2	8.0
EN	5.8	6.0	6.1	6.0	5.8	5.4	6.1	6.0	5.9	5.6	5.9
IO	7.1	5.4	5.5	5.8	5.5	4.9	5.7	5.6	5.3	4.8	6.1
EL	0.7	1.1	1.5	1.5	1.2	1.2	1.0	1.2	1.3	1.4	1.4
IN	2.4	2.3	2.4	2.2	2.0	2.0	2.3	2.2	2.2	2.1	2.3

#### Description of holotype

**(Figs 5, 6).** Adult male SVL 69.5 mm; head moderate in length (HL/SVL 0.27), width (HW/HL 0.68), depth (HD/HL 0.38), distinct from neck, triangular in dorsal profile; lores slightly concave anteriorly, weakly inflated posteriorly; prefrontal region weakly concave; canthus rostralis rounded; snout elongate (ES/HL 0.42), rounded in dorsal profile; eye large (ED/HL 0.21); ear opening obliquely elliptical, small; eye to ear distance greater than diameter of eye; rostral rectangular, divided by a dorsal furrow, bordered posteriorly by large left and right supranasals and one smaller azygous internasal, bordered laterally by first supralabials; external nares bordered anteriorly by rostral, dorsally by large supranasal, posteriorly by two unequally sized smaller postnasals, bordered ventrally by first supralabial; 9R/9L rectangular supralabials, second through seventh supralabials nearly same size as first, then tapering below eye; 9R/9L infralabials tapering smoothly to just below and slightly past posterior margin of eye; scales of rostrum and lores flat to slightly domed, larger than granular scales on top of head and occiput; scales of occiput intermixed with distinct, small tubercles; superciliaries subrectangular, largest anterodorsally; mental triangular, bordered laterally by first infralabials and posteriorly by large left and right trapezoidal postmentals contacting medially for 50% of their length posterior to mental; one row of enlarged, square to rectangular sublabials extending posteriorly to first (L) and fourth (R) infralabial; gular and throat scales small, granular, grading posteriorly into slightly larger, flatter, smooth, imbricate, pectoral, and ventral scales.

Body relatively short (AG/SVL 0.46) with well-defined denticulate ventrolateral folds; dorsal scales small, granular interspersed with larger, conical, semi-regularly arranged, weakly keeled tubercles; tubercles extend from occipital region onto base of tail and slightly beyond as paravertebral rows; smaller tubercles extend anteriorly onto nape and occiput, diminishing in size anteriorly; approximately 19 longitudinal rows of tubercles at midbody; approximately 20 paravertebral tubercles; small tubercles on flanks; 42 longitudinal rows of flat, imbricate, ventral scales much larger than dorsal scales; 158 transverse rows of ventral scales; 13 large, pore-bearing, precloacal scales; no deep precloacal groove or depression; and three rows of enlarged post-precloacal scales on midline.

Forelimbs moderate in stature, relatively short (HumL/SVL 0.13; ForL/SVL 14.7); granular scales of forearm larger than those on body, interspersed with slightly larger tubercles; palmar scales rounded, slightly raised, subimbricate; digits well-developed, relatively short, inflected at basal interphalangeal joints; digits narrower distal to inflections; subdigital lamellae wide, transversely expanded proximal to joint inflections, narrower lamellae distal to inflections; claws well-developed, claw base sheathed by a dorsal and ventral scale; 9R/9L expanded and 11R/11L unexpanded lamellae beneath the fourth finger; hind limbs longer and thicker than forelimbs, moderate in length (FemL/SVL 18.4; TibL/SVL 0.09), covered dorsally by granular scales interspersed with moderately sized, conical tubercles dorsally and posteriorly and anteriorly by flat, larger, subimbricate scales; ventral scales of thigh flat, imbricate, larger than dorsals; subtibial scales flat, imbricate; one row of 8R/8L enlarged pore-bearing femoral scales not continuous with enlarged pore-bearing precloacal scales, terminating distally at knee; proximal femoral scales smaller than distal femorals, the former forming an abrupt union with much smaller, rounded, ventral scales of posteroventral margin of thigh; plantar scales raised, subimbricate; digits relatively long, well-developed, inflected at basal interphalangeal joints; 9R/9L wide, transversely expanded subdigital lamellae on fourth toe proximal to joint inflection extending onto sole, and 11R/11L unexpanded lamellae beneath the fourth toe distal to joint inflection; and claws well-developed, claw base sheathed by a dorsal and ventral scale.

Posterior one-fifth of tail regenerated, 83.0 mm long (TL/SVL 1.19), 6.5 mm in width at base, tapering to a point; nearly square in cross-section; dorsal scales flat, intermixed with large tubercles forming spinose paravertebral rows; row of large dorsolateral caudal tubercles; large, posteriorly directed, spinose tubercles forming wide ventrolateral caudal fringe; much larger scales of ventrolateral fringe occur at regular intervals; medial subcaudals slightly enlarged, no enlarged single medial subcaudal longitudinal row; subcaudals, larger than dorsal caudals; small longitudinal ventrolateral subcaudal ridges; base of tail bearing hemipenal swellings; 3R/3L conical postcloacal tubercles at base of hemipenal swellings; and postcloacal scales flat, imbricate.

#### Coloration in life

**(Fig. 4).** Ground color of the head body, limbs, and tail tan; diffuse darker mottling on the top of the head; wide, cream-colored postorbital stripe extends across the occipital region from one eye to the other; faint whitish canthal markings; dark-brown, nuchal band bearing two posteriorly directed projections; paired dark-brown paravertebral blotches between forelimb insertions; three wide, dark brown, irregularly shaped and deeply emarginated body bands edged in slightly darker brown between the limb insertions; band interspaces bearing irregularly shaped scattered dark-brown markings; limbs generally unicolor tan; digits darkly banded; six wide pale-brown caudal bands separated by six paler colored bands; bands do not encircle tail; ventral surfaces of body and limbs beige, generally immaculate, subcaudal region generally darker; iris orange-gold in color bearing black vermiculations.

#### Distribution.

*Cyrtodactylusdenticulatus* sp. nov. represents the northwestern-most species of the *brevipalmatus* group. At present, it is known only from the type locality at Chao Doi waterfall, Tha Song Yang District, Tak Province, western Thailand (Fig. 1).

#### Etymology.

The specific epithet *denticulatus* is given as a noun in apposition, meaning “denticulate” or with small teeth, a reference to bearing small tooth-like dorsolateral and ventrolateral caudal tubercles and denticulate ventrolateral body folds.

#### Comparisons of categorical data

**(Tables 2–4).***Cyrtodactylusdenticulatus* sp. nov. can be separated from all other species of the *brevipalmatus* group by having denticulate ventrolateral body folds and small ventrolateral subcaudal ridges. These characters were not observed in any other individuals of the *brevipalmatus* group (*n* = 51). The presence or absence of the following characters showed no intrapopulational variation. *Cyrtodactylusdenticulatus* sp. nov. can be separated from *C.brevipalmatus*, C.cf.ngati1, C.cf.ngati2, *C.fluvicavus*, *C.interdigitalis*, *C.ngati*3, *C.rukhadeva*, and *C.* sp. 13 by have large as opposed to small dorsolateral caudal tubercles and a ventrolateral caudal fringe. It differs from C.cf.ngati1, C.cf.ngati2, *C.interdigitalis*, *C.ngati*3, *C.rivularis*, *C.rukhadeva*, *C.* sp. 11, and *C.* sp. 13 by having ventrolateral heterogeneous ventrolateral caudal fringe scales. It differs from all species except *C.kochangensis*, *C.rivularis*, *C.rukhadeva*, *C.* sp. 11, and *C.thongphaphumensis* by having a square cross-section of the tail. It differs from all species except *C.brevipalmatus*, C.cf.ngati1, C.cf.ngati2, *C.fluvicavus*, *C.ngati*, *C.ngati*3, *C.thongphaphumensis* and *C.uthaiensis* in having slightly enlarged medial subcaudal scales. *Cyrtodactylusdenticulatus* sp. nov. can be differentiated from *C.interdigitalis*, *C.rivularis*, and *C.rukhadeva* by lacking an enlarged longitudinal row of medial subcaudals. From *C.interdigitalis* and *C.uthaiensis* it differs by lacking intermittently enlarged, medially furrowed, and posteriorly emarginate medial subcaudals. Being that this species is known from only one specimen we acknowledge that some of the meristic differences in Tables 2–4 may eventually prove not be diagnostic with the addition of more specimens just as other meristic characters may prove to be statistically diagnosable. Until then, we refrain from including metric characters in the diagnosis. Potential diagnostic meristic characters are highlighted (Tables 2–4).

#### Natural history.

**Figure 7.**
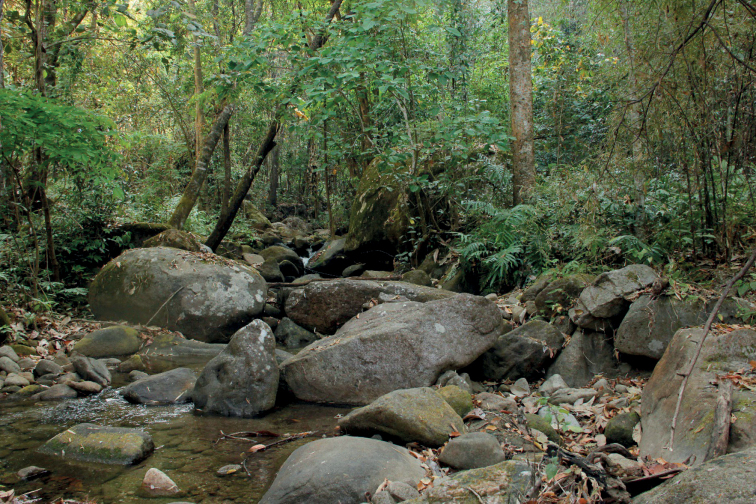
Habitat of *Cyrtodactylusdenticulatus* sp. nov. AUP-00680 from the Chao Doi waterfall, Mae Meoi, Tha Song Yang District, Tak Province, Thailand.

The holotype was collected at night between 19.00–23.00 hours in a bamboo forest near a rocky stream. The lizard was found on a bamboo branch 4 m above a large granite boulder (Fig. 7). The habitat was composed of large Bamboo (*Dendrocalamuscopelandii*) Myrtaceae (*Syzygium* sp.) and dipterocarp trees (*Anisopteracostata*) (Fig. 5). We speculate *C.denticulatus* sp. nov. is an arboreal specialist that generally resides in the upper canopy similar to *C.brevipalmatus* and *C.elok* (Grismer 2011). At the type locality, *C.denticulatus* sp. nov. was recorded in sympatry with *Cyrtodactylus* sp. 1 (Chomdej et al. 2021), *Trimeresurusguoi*, *Ansoniainthanon*, *Rhacophorusrhodopus*, and *Thelodermaalbopunctatum*.

## ﻿Discussion

The data above clearly demonstrate the unique phylogenetic and morphological properties of the individual described above as *Cyrtodactylusdenticulatus* sp. nov. However, describing a species on the basis of a single specimen can be potentially misleading, especially in the absence of genetic data. Here we provide compelling genetic evidence for the unique phylogenetic position of this specimen wherein it resides on its own long branch (pairwise sequence divergence of 7.87–21.94%) that is not embedded within that of any other species nor sister to any other species as with *C.kochangensis* Grismer, Aowphol, Yodthong, Ampai, Temprayon, Aksornneam & Rujirawan, 2022 and *C.uthaiensis* Grismer, Aowphol, Yodthong, Ampai, Temprayon, Aksornneam & Rujirawan, 2022. Its phylogenetic position is what delimits it as a distinct evolutionarily independent lineage, not its morphology. The morphological diagnosis of a species provides evidence of how different or similar a species may be to other closely related species but has no bearing on whether or not it is a new species (see discussion in Grismer et al. 2022b). Many cryptic species defy discrete morphological diagnoses, as the designation of “cryptic” implies. The morphological diagnosis of a species based on a single specimen is incomplete, as it does not capture the range of variation of the species of which it represents. However, given the general difficulty in finding cryptic arboreal species, waiting for the acquisition of additional material that does not bear on its species status, in our opinion, is not the best option. A formal description of the species can begin the process of levying legal conservation measures and does not relegate undescribed species as “ecological ghosts” with no legal protection. This is particularly germane to tropical upland endemics whose ecosystems are some of the most imperiled in the world.

The addition of *Cyrtodactylusdenticulatus* sp. nov. brings the total number of described species in the *brevipalmatus* group to ten. Based on photographs in social media, there are as many as five potentially undescribed species from unsampled areas (Fig. 1). Given that the members of this group are cryptic species generally restricted to isolated upland habitats, we are reasonably certain there are more populations to be discovered and described. This becomes particularly important in this era of climate change where some of its greatest negative impacts occur in tropical upland ecosystems.

## Supplementary Material

XML Treatment for Cyrtodactylus denticulatus

## Supplementary materials

Supplementary material 1 Dataset of meristic, normalized morphometric, and categorical characters Data type table (.csv file) https://binary.pensoft.net/file/855854This dataset is made available under the Open Database License (http://opendatacommons.org/licenses/odbl/1.0/). The Open Database License (ODbL) is a license agreement intended to allow users to freely share, modify, and use this Dataset while maintaining this same freedom for others, provided that the original source and author(s) are credited.Siriwadee Chomdej, Chatmongkon Suwannapoom, Waranee Pradit, Apichaya Phupanbai, L. Lee Grismer
